# Naturally Occurring Incompatibilities between Different *Culex pipiens pallens* Populations as the Basis of Potential Mosquito Control Measures

**DOI:** 10.1371/journal.pntd.0002030

**Published:** 2013-01-31

**Authors:** Lin Chen, Changliang Zhu, Donghui Zhang

**Affiliations:** 1 Department of Pathogen Biology, Nanjing Medical University, Nanjing, Jiangsu, P. R. China; 2 Jiangsu Province Key Laboratory of Modern Pathogen Biology, Nanjing Medical University, Nanjing, Jiangsu, P. R. China; Monash University, Australia

## Abstract

**Background:**

Vector-borne diseases remain a threat to public health, especially in tropical countries. The incompatible insect technique has been explored as a potential control strategy for several important insect vectors. However, this strategy has not been tested in *Culex pipiens pallens*, the most prevalent mosquito species in China. Previous works used introgression to generate new strains that matched the genetic backgrounds of target populations while harboring a new *Wolbachia* endosymbiont, resulting in mating competitiveness and cytoplasmic incompatibility. The generation of these incompatible insects is often time-consuming, and the long-term stability of the newly created insect-*Wolbachia* symbiosis is uncertain. Considering the wide distribution of *Cx. pipiens pallens* and hence possible isolation of different populations, we sought to test for incompatibilities between natural populations and the possibility of exploiting these incompatibilities as a control strategy.

**Methodology/Principal Findings:**

Three field populations were collected from three geographic locations in eastern China. Reciprocal cross results showed that bi-directional patterns of incompatibility existed between some populations. Mating competition experiments indicated that incompatible males could compete with cognate males in mating with females, leading to reduced overall fecundity. F1 offspring from incompatible crosses maintained their maternal crossing types. All three populations tested positive for *Wolbachia*. Removal of *Wolbachia* by tetracycline rendered matings between these populations fully compatible.

**Conclusions/Significance:**

Our findings indicate that naturally occurring patterns of cytoplasmic incompatibility between *Cx. pipiens pallens* populations can be the basis of a control strategy for this important vector species. The observed incompatibilities are caused by *Wolbachia*. More tests including field trials are warranted to evaluate the feasibility of this strategy as a supplement to other control measures.

## Author Summary

Population suppression is an important component of mosquito control measures. The incompatible insect technique exploits the monogamous mating behavior of female mosquitoes to decrease the percentage of females inseminated by compatible males and hence reduce overall fecundity. Previous studies used genetically engineered *Wolbachia*-infected mosquitoes as the sources of incompatible males. The long-term stability of these mosquitoes is unknown. In this study, we examined naturally occurring incompatibilities between different field populations of *Culex pipiens pallens*, a vector of West Nile Virus and filarial worms widely distributed in China. We found that bi-directional patterns of incompatibility existed between some *Cx. pipiens pallens* populations. Incompatible males could compete against cognate males in mating with females and suppress reproduction. The level of suppression depended on the relative population sizes of incompatible and cognate males. We also found that these incompatibilities were preserved in the offspring from incompatible crosses, indicating that this control strategy is likely to be sustainable when applied repeatedly to successive mosquito generations. Our data also indicate that these incompatibilities were caused by endosymbiont *Wolbachia*, which is also the basis for cytoplasmic incompatibility in a variety of mosquito species. Our results may help to design simpler and more time-effective control strategies for a number of vector-borne diseases.

## Introduction

Vector-borne diseases especially those transmitted by mosquitoes such as malaria, dengue fever, Japanese encephalitis, West Nile fever, Chikungunya and lymphatic filariasis are still important scourges responsible for millions of deaths each year, especially in tropical and developing countries. The lack of effective vaccines for major mosquito-borne diseases and the development of resistance to available chemotherapies both underscore the importance of reducing the numbers of major vector mosquitoes. Insecticides are the major weapons for mosquito control. Earlier efforts using insecticides to reduce malaria and other neglected tropical diseases had been met with success [1], [2]. But, long-term intensive use of insecticides has led to the development of insecticide resistance in important vector mosquito species, jeopardizing the effectiveness of insecticide-based vector control [3]. In addition, the adverse effects of insecticides on human health and the environment could not be ignored [4], [5].

With these concerns, biological approaches are called upon as alternatives to chemical control. One approach is to render the arthropods incapable of disease transmission. This strategy uses transgenesis or paratransgenesis to introduce foreign genes into arthropod populations. The expression of the transgenes makes these arthropods resistant to infections or unsuitable for parasite development to their infective stages [6], [7]. Introduction of endosymbionts such as *Wolbachia*, maternally inherited obligatory intracellular bacteria, into certain mosquito species can also decrease their vectorial capacity and have yielded novel strategies for population replacement [8]–[11]. Another approach resembles chemical control in that it aims at population reduction. For example, transgenes or biological agents such as *Wolbachia* can be introduced into mosquito populations to reduce fertility [12]–[15].

Because females of many insect species mate only once in their life-time, unproductive mating effectively precludes their reproduction or reduces their fecundity. Two strategies have been developed to take advantage of their monogamous behaviors, namely the sterile insect technique (SIT) and the incompatible insect technique (IIT). SIT uses radiologically or chemically sterilized males to suppress target populations. IIT uses incompatibilities especially cytoplasmic incompatibility (CI) between males and females to reduce female fecundity. CI is an incompatibility between a sperm and an egg that interferes with the normal development of a zygote. It is caused by endosymbionts in the cytoplasm such as *Wolbachia* [15]–[17]. Since their initial identification in the ovaries of *Culex* mosquitoes [18], *Wolbachia* have been found in the reproductive tissues of the majority of tested arthropod species [19]. *Wolbachia* cause a variety of reproductive abnormalities besides CI, such as feminization of genetic males, thelytokous parthenogenesis (female offspring being produced from unfertilized eggs), and male-killing [15], [20]. Among these abnormalities, CI has been explored as a potential tool for the development of novel and environmentally friendly strategies for insect controls [9], [21]. Because CI exists between sperm of *Wolbachia*-infected mosquitoes and eggs of an uninfected or an incompatible strain of *Wolbachia*-infected mosquitoes, CI-based IIT is applicable to both *Wolbachia*-negative and *Wolbachia*-positive target populations. CI prevents embryonic development, thus making the mating events unproductive. Consequently, those mated females are unable to reproduce or have their fecundity greatly reduced.

The application of CI to suppress insect populations predated the identification of *Wolbachia* as its causative agent. Laven used an introgressed *Culex pipiens fatigans* strain with maternal cytoplasm derived from a Paris strain to successfully suppress the incompatible local *Cx. pipiens fatigans* populations in Myanmar [22]. Subsequently, CI-based suppression was trialed for the European cherry fruit fly *Rhagoletis cerasi* [23] and almond warehouse moth *Cadra* (*Ephestia*) *cautella* [24]. To improve the safety of this suppression strategy, an irradiation step was included in several studies on *Culex* mosquitoes to minimize inadvertent release of fertile females and minority-type (compatible) males [25]–[27]. Medfly *Ceratitis capitata* transinfected with *Wolbachia* derived from *R. cerasi* have been generated for IIT approaches to suppress medfly populations [28]. CI has also been utilized to develop strategies to control *Aedes* mosquitoes. Through interspecific hybridization and introgression, an *Aedes polynesiensis* strain harboring *Wolbachia* derived from *Aedes riversi* was generated. This new strain is bi-directionally incompatible with the natural strain [29].

*Wolbachia*-infected mosquito strains that display novel patterns of CI for population suppression are usually created by introgression or transinfection [30]–[32]. The preparation of incompatible mosquitoes using such methods is often laborious and time-consuming. Because introgression involves putting *Wolbachia* into new host genetic backgrounds, the long-term stability of such novel mosquito-*Wolbachia* symbioses is unknown. In addition, although IIT has been applied to a variety of insects, it has not been tested in *Culex pipiens pallens*, one of the most prevalent mosquito species in China. In this study, using three *Cx. pipiens pallens* field populations collected in eastern China, we tested the applicability of exploiting incompatibilities between naturally occurring populations to suppress target populations. We report that incompatible males could compete with cognate males in mating with females, resulting in reduced fecundity. Though the incompatibilities were not absolute, they were preserved with hybrid offspring keeping the maternal crossing types. Our data indicate that this strategy may be a viable and sustainable approach over time to suppress populations of *Cx. pipiens pallens*.

## Materials And Methods

### Mosquitoes

**Figure 1:**
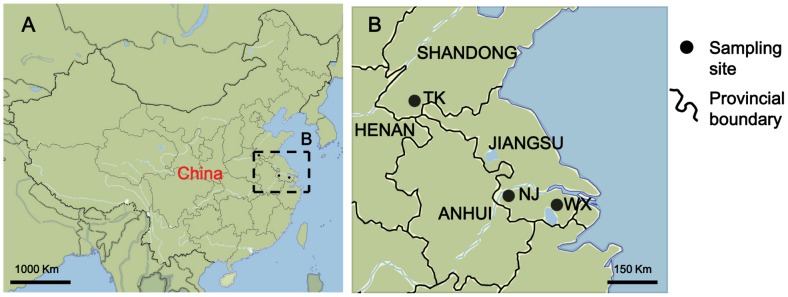
Collecting sites of *Cx. pipiens pallens* field populations. (A) The mosquito populations were collected from eastern China; (B) Three collecting sites Tangkou, Nanjing and Wuxi are shown in detail. The mosquito populations collected are designated TK, NJ and WX, respectively.

WX (Wuxi, Jiangsu Province, 31°33′58.47″N, 120°18′9.88″E), NJ (Nanjing, Jiangsu Province, 32°3′30.11″N, 118°47′47.28″E), and TK (Tangkou, Shandong Province, 34°52′34.97″N, 117°22′53.69″E) populations of *Cx. pipiens pallens* were used in this study (Figure 1) [33]. WX and TK populations were collected in 2008 from July to August. NJ population was collected in July, 2006. For each population, several hundreds of larvae were collected with 350-ml dippers from over 50 larval habitats in public areas of the respective location. Fourth-instar larvae were identified to species by morphology, including the aspect ratio of the air tube and the number of pectens and tufts on it. The larvae were then brought back and reared in the insectary. Mosquitoes were kept at 28°C and 75% relative humidity with 14 h∶10 h light∶dark cycle. Adult mosquitoes were fed 10% (w/v) glucose solution prior to blood meals.

### Crossing experiments

To separate virgin females and males, pupae from each population were put into 15-ml tubes with water for individual emergence. Afterward, male and female adults were raised in 30.5×30.5×30.5 cm cages. Females 2 days after emergence and males 3 days after emergence were used in mating experiments. Each set of crossings included combination groups of virgin males and females from two different *Cx. pipiens pallens* populations, with combinations of males and females from the same populations as positive controls. Females and males placed in the same cages were given 2 days to mate. Females were blood fed after mating, then the egg rafts were given 48 hours after oviposition to hatch in separate containers. The numbers of eggs and larvae were counted under stereoscope and the hatching rates (HR) for individual rafts were calculated. Unhatched egg rafts were all examined for fertilization through microscopic observation of embryonic development [34]. Each experiment was performed twice, shown here is a representative result.

### Detection of *Wolbachia*

Total DNA of individual *Cx. pipiens pallens* mosquito was extracted using a method previously described [35]. Polymerase chain reaction (PCR) was first carried out using generic primers to amplify *wsp* gene which encodes the major surface protein of *Wolbachia* to determine the infection status of the mosquito [36]. Studies have reported that the majority of insect *Wolbachia* belong to supergroups A and B, specific *wsp* primers were used accordingly for typing [37]. All primers were adopted from published studies. Primers wF and wR (see Table 1 for primer sequences) were used to amplify a 590–632 bp fragment from all *Wolbachia* strains; primers wAF and wR were used to amplify a 556 bp fragment characteristic of supergroup A; primers wBpipF and wR were used to amplify a 501 bp fragment characteristic of *w*Pip strains of supergroup B; and primers wBcauBF and wR were used to amplify a 466 bp fragment characteristic of the *w*CauB strains of supergroup B. In addition, PCR was carried out to amplify the ankyrin domain-encoding gene *ank2* to differentiate *w*Pip strains into groups I-V based on 313–511 bp amplification fragments [38].

**Table 1:** Genes used for *Wolbachia* typing and their PCR amplification primers.

Gene	Putative product	Primer (5′-3′)	Size of expected amplification fragment (bp)	References
*wsp*	major surface protein	wF-TGGTCCAATAAGTGATGAAGAAAC	590–632 bp	[36], [37]
		wAF-TGAAATTTTACCTCTTTTC	556 bp	
		wBpipF-AAGGAACCGAAGTTCATG	501 bp	
		wBcauBF-CCATCTTTTCTAGCTGGA	466 bp	
		wR-AAAAATTAAACGCTACTCCA		
*ank2*	ankyrin domain protein	F-CTTCTTCTGTGAGTGTACGT	313–511 bp	[38]
		R2-TCCATATCGATCTACTGCGT		

All PCR reactions were composed as follows: 1 unit of Pyrobest Taq DNA polymerase (Takara, Japan), 5 µl 10× Pyrobest Buffer II (Mg^2+^ Plus), 4 µl of 2.5 mM dNTPs, 5 ng total DNA, 2 µl of each 10 µM primer, and ddH_2_O was added to bring up the total volume to 50 µl. The amplification of *wsp* gene was performed as follows: 32 cycles of 94°C for 30 s, 55°C for 30 s and 72°C for 60 s, followed by a final extension at 72°C for 7 min. The amplification of *ank2* gene was performed as follows: 35 cycles of 94°C for 30 s, 52°C for 30 s and 72°C for 60 s, followed by a final extension at 72°C for 7 min. PCR products were resolved by 1% agarose gel electrophoresis and stained with ethidium bromide. PCR products of ten mosquitoes from each population were used for sequencing of *wsp* and *ank2* alleles by chain-termination method (Invitrogen). Sequence analysis was carried out using DNAMAN software. The unique DNA sequences were deposited into GenBank (accession numbers JX050182 - JX050187).

### Tetracycline treatment and fluorescence staining

Tetracycline treatment to eliminate *Wolbachia* from *Culex* populations was carried out according to published methods [17]. Tetracycline (Amresco) at a concentration of 0.05 mg/ml was used for the treatment through both larval and pupal stages. Eggs were placed on tetracycline water solution to hatch. Surviving larvae were transferred to fresh tetracycline solution every 24 hours. A normal infusion was prepared in parallel and fed to larvae in tetracycline solution. The elimination of *Wolbachia* was checked by Hoechst 33342 (Sigma) staining [39]. Egg rafts within 1 hour of oviposition were placed in 5 ml fixation buffer (182 mM KCl, 46 mM NaCl, 3 mM CaCl_2_, 10 mM Tris pH 7.2, 3.7% formaldehyde) and overlaid with 5 ml *n*-heptane in a 50-ml tube. After incubation at room temperature for 15 min with constant shaking, fixation buffer was replaced with 10 ml methanol. The eggs were incubated at room temperature for 10 min with constant shaking. The *n*-heptane layer was replaced with 10 ml methanol. The eggs were washed for 2 times with methanol, and stained with Hoechst 33342 (1 µg/ml in PBS) for 15 min before visualization under microscope.

### Statistical analysis

Statistical differences in hatching rate among crossing groups were examined using the Student's *t*-test. Linear regression analysis was conducted to determine the correlation coefficient between hatching rate and percentage of incompatible males. All statistical analyses were carried out using SPSS Statistics 17.0.

## Results

### Incompatibility exists between *Cx. pipiens pallens* populations

**Figure 2:**
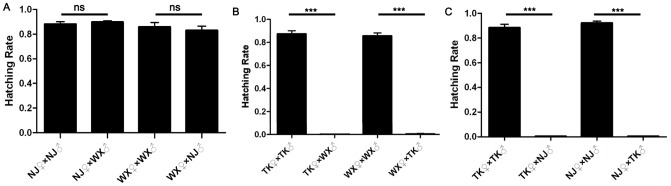
Reproductive compatibilities between three *Cx. pipiens pallens* field populations. Three *Cx. pipiens pallens* field populations NJ, WX and TK were used in reciprocal crosses to test their reproductive compatibilities. For each cross, an equal number of virgin females and males were kept in a cage for 48 hours before blood feeding. After oviposition, egg rafts were collected and allowed to hatch. The average hatching rates of these crosses were compared. (A) Mating combinations of NJ and WX populations, (B) Mating combinations of TK and WX populations, (C) Mating combinations of NJ and TK populations. Error bars represent standard errors; ns, non-significant *P*-value; *** *P*<0.0001 by *t*-test.

We first tested the compatibilities between available *Cx. pipiens pallens* populations collected from three geographic locations in China. Reciprocal crosses among these three field populations were performed as outlined in Table S1. In the mating combinations of NJ and WX populations, no significant incompatibility was detected in either NJ♀×WX♂ or WX♀×NJ♂ cross as compared to NJ♀×NJ♂ and WX♀×WX♂ control groups (Table S1 and Figure 2A). In the mating combinations of TK and WX populations, bi-directional incompatibility was observed, with both TK♀×WX♂ and WX♀×TK♂ crosses having significantly reduced average hatching rate as compared to TK♀×TK♂ and WX♀×WX♂ control groups (Table S1 and Figure 2B). In the WX♀×TK♂ group, 14 larvae hatched out of 21 egg rafts, making the average hatching rate 0.005±0.026. The incompatibility was more pronounced in the TK♀×WX♂ group, with 2 larvae hatched out of a total of 18 egg rafts, resulting in an average hatching rate of 0.001±0.001. In comparison, the hatching rates in the control groups TK♀×TK♂ and WX♀×WX♂ were 0.873±0.028 and 0.856±0.003, respectively.

Similarly, bi-directional incompatibility was detected in the mating combinations of NJ and TK populations (Table S1 and Figure 2C). The average hatching rates for TK♀×NJ♂ and NJ♀×TK♂ crosses were 0.004±0.003 and 0.004±0.002, respectively. In comparison, the average hatching rates were 0.883±0.028 and 0.922±0.015 for TK♀×TK♂ and NJ♀×NJ♂ control groups, respectively.

### The incompatibility is not caused by mating failure

The observed bi-directional incompatibility between TK and WX populations and between TK and NJ populations could be a result of mating failure or some post-mating events. To distinguish between these two possibilities, insemination status and embryonic development in incompatible crosses were examined. The female genitalia of *Culex* mosquitoes include three oval-shaped spermathecal capsules for spermatozoa storage which are connected to vagina through spermathecal ducts. During coitus, some or all of these capsules are filled with seminal fluid from a male and become inflated. The females from incompatible crosses were examined and compared to virgin females. Because three spermathecal capsules from the same individual female had variable sizes before insemination as seen in virgin females, and it was likely that they also varied from mosquito to mosquito, inflation was not always a reliable indicator of successful insemination. Instead, the presence of spermatozoa inside the spermathecal capsules or ovarioles was checked. In all crossing experiments we conducted, the females from both TK♀×NJ♂ and TK♀×WX♂ crosses contained spermatozoa, indicating that they had mated. In addition, the egg rafts were examined 48 hours after oviposition. The eggs from incompatible crosses showed clear embryonic development (Figure S1). Inside the eggs from TK♀×WX♂ cross, there was segment formation along the axis. At the anterior end of the eggs, two red spots representing primitive eyes (stemmata) were visible. Consistent with previous reports, these embryos showed some abnormalities [34]. One evident difference between TK♀×WX♂ cross and compatible crosses was that the incompatible embryos had disorientated bristles. In the eggs from TK♀×NJ♂ cross, similar development was observed. 1–3 (mostly 2) pigmented stemmata were formed. Most stemmata were located in the head region, but some did not seem to have a specific localization. In some eggs, two stemmata were aligned anteroposteriorly instead of being side-by-side. In addition, there was segment formation along the axis in some eggs. Disoriented bristles were also observed in the eggs from TK♀×NJ♂ cross. These observations further confirmed that the females in the incompatible crosses had mated. For those females needed for subsequent mating experiments, only embryonic development inside the eggs was checked to confirm their insemination status.

### Mating with incompatible males prevents females from subsequent mating with cognate males

**Figure 3:**
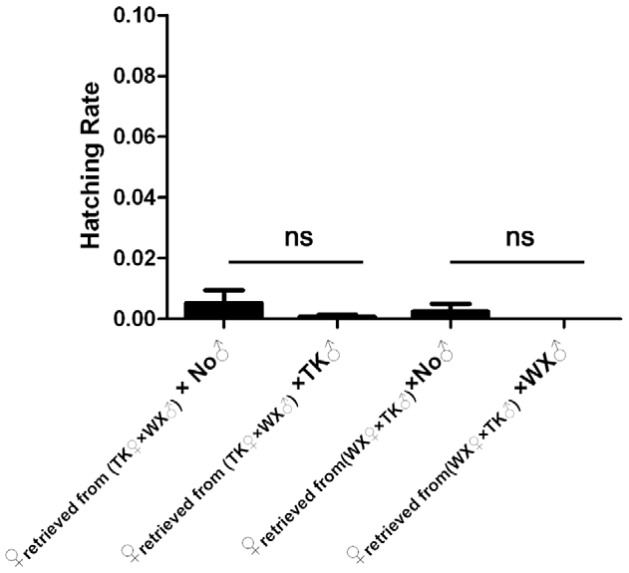
Effect of subsequent encounter with cognate males on the fecundity of the females retrieved from incompatible crosses. Females were retrieved from the two incompatible crosses as shown in Figure 2B. TK or WX females were equally divided into two subgroups, with one mixed with cognate males and the other kept alone (no male). After a second blood feeding and oviposition, egg rafts were collected and allowed to hatch. The average hatching rates of these subgroups were compared. Error bars represent standard errors; ns, non-significant *P*-value.

Female monogamy in insects is common but often not absolute. Considering these populations were geographically isolated, it was possible that mating with incompatible males did not preclude subsequent mating of the inseminated females. To test if mating with incompatible males made the females refractory to re-mating, females were retrieved from the incompatible crosses and tested for their ability to mate with cognate males. In the aforementioned crossing experiment, the combination of TK and WX populations displayed higher level of incompatibility than that of TK and NJ populations, so TK and WX populations were chosen in subsequent experiments. TK♀ from TK♀×WX♂ cross and WX♀ from WX♀×TK♂ cross were separated from WX♂ and TK♂, respectively. Each group was equally divided into two subgroups, with one subgroup mixed with cognate males and the other kept alone (Table S2). If subsequent mating could happen, the subgroup mixed with cognate males would become inseminated with both compatible and incompatible spermatozoa and result in higher hatching rates than the subgroup kept separate from cognate males. As shown in Figure 3 and Table S2, the hatching rates in TK♀ subgroup mixed with TK♂ and in TK♀ subgroup kept alone without males were not significantly different (*t* = −1.013, df = 18, *P* = 0.324). Similarly, the hatching rates in WX♀ subgroup mixed with WX♂ and in WX♀ subgroup kept alone without males were not significantly different (*t* = −1.0, df = 6, *P* = 0.356). These results indicate that both TK♀ and WX♀ became refractory to subsequent mating after they mated with incompatible males.

### Incompatible males can competitively decrease females mating with compatible males and reduce overall fecundity in a dose-dependent manner

Because these incompatible females and males can successfully mate in the absence of compatible males and produce reduced numbers of offspring, we then tested if incompatible mating would still occur when compatible males were available. We also tested if the reduction in fecundity depended on the number of incompatible males introduced. To that end, two sets of experiments were performed using TK and WX females (Table S3). In each set, an equal number of females were placed in different cages together with no male (blank), equal number of compatible males (positive control), equal number of compatible males plus equal number of incompatible males, or equal number of males plus 3× as many incompatible males. In addition, as in the previous experiment, 48 hours after the first oviposition, egg rafts were checked for embryonic development to ascertain that the females were inseminated. Subsequently, females were collected from each group and divided into two subgroups to be mixed with either no male or cognate males (Table S4). The hatching rates from the second oviposition were compared to determine if mating with mixed male populations made these females refractory to subsequent mating even when only cognate males were available.

**Figure 4:**
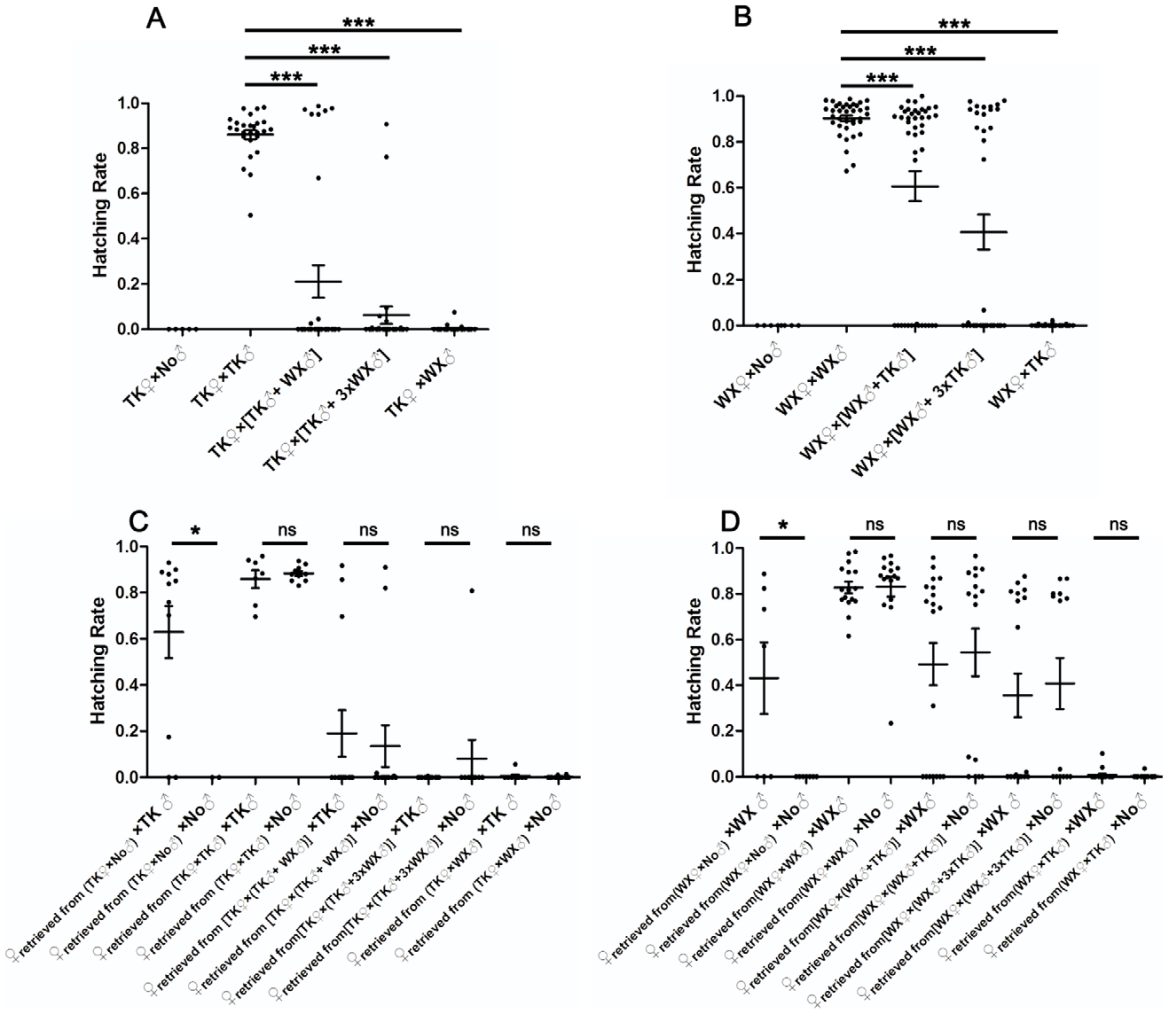
Incompatible males competitively suppress reproduction in a dose-dependent manner. Females were mixed with cognate males and an increasing number of incompatible males. They were given 48 hours before blood feeding. The average hatching rates of these crosses were compared. Subsequently, females were retrieved and divided equally into two subgroups, with one mixed with cognate males and the other kept alone. The average hatching rates of these subgroups were compared. (A) TK♀ mixed with TK♂ and WX♂; (B) WX♀ mixed with WX♂ and TK♂; (C) TK♀ retrieved from A were mixed with TK♂ or kept alone; (D) WX♀ retrieved from B were mixed with WX♂ or kept alone. Error bars represent standard errors; ns, non-significant *P*-value; *** *P*<0.0001; * *P*<0.05 by *t*-test.

The results (Table S3, Figure 4A) show that in the positive control group (TK♀×TK♂) the average hatching rate was 0.861±0.020. When an equal number of WX males were included in the cage [TK♀×(TK♂+WX♂)], the average hatching rate dropped to 0.211±0.071. This value was further decreased to 0.063±0.039 in the group that included 3× as many WX males [TK♀×(TK♂+3×WX♂)]. In comparison, the average hatching rate of TK♀ mixed with only WX♂ was 0.004±0.003 and TK♀ kept alone produced no larva. These data indicate TK♀ mated with TK♂ or WX♂ in the presence of mixed male population, i.e., even in the presence of TK♂, mating between TK♀ and WX♂ still occurred, which resulted in reduced overall fecundity of the groups. The extent of fecundity reduction correlated with the ratio of WX♂ to TK♂.

Similarly, in parallel experiment with WX♀ (Table S3, Figure 4B), the average hatching rate of WX♀ was reduced from 0.903±0.013 in the positive control group (WX♀×WX♂) to 0.607±0.066 in the group with an equal number of competing TK♂ included [WX♀×(WX♂+TK♂)] and further to 0.407±0.077 in the group with 3× competing TK♂ included [WX♀×(WX♂+3×TK♂)]. The average hatching rate of WX♀ mixed with only TK♂ (WX♀×TK♂) was 0.001±0.001. WX♀ kept alone produced no larva. These data also indicate that the frequency of WX♀×TK♂ mating in the presence of mixed male population can also be increased with an increasing TK♂∶WX♂ ratio.

As shown in Figure 4C, after retrieved TK♀ were mixed with TK♂ or no male, these two subgroups produced similar hatching rates in their second oviposition. Similarly, subsequent mixing of retrieved WX♀ with WX♂ did not significantly increase the hatching rates in the second oviposition compared to the subgroups of retrieved WX♀ kept alone (Figure 4D). These results indicate both TK♀ and WX♀ became refractory to subsequent mating after they mated with mixed male populations. This was similar to the scenario in which their first mating was with incompatible males only. Polyandry was not common or completely absent for these female populations. In addition, the average hatching rates in their subsequent ovipositions correlated with the hatching rates in their first oviposition. A higher number of incompatible males during the first mating event resulted in lower fecundity during the first and subsequent gonotrophic cycles.

**Figure 5:**
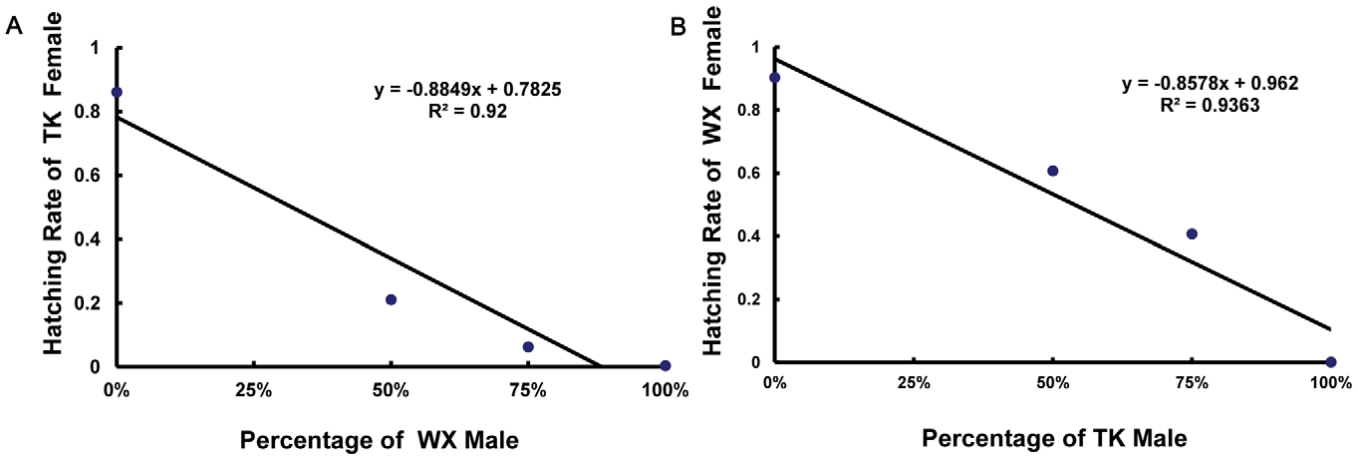
Correlation between group average hatching rate and the percentage of incompatible males. The average hatching rate of a group was plotted against the percentage of incompatible males which was calculated by dividing the number of incompatible males by the total number of males (incompatible and cognate). (A) TK females with TK and WX males; (B) WX females with WX and TK males.

The correlation between hatching rate and the proportion of incompatible males was plotted for both TK♀ and WX♀. As shown in Figure 5, based on the curves, the fecundity of TK♀ could be diminished when inundated by excess WX♂. 9× WX♂ can achieve nearly complete suppression of TK♀ fecundity. On the other hand, the maximal fecundity reduction of WX♀ by TK♂ is around 90% in one generation. The r^2^ value is 0.92 (*P*<0.05) for TK♀ curve (Figure 5A), and 0.9363 (*P*<0.05) for WX♀ curve (Figure 5B).

### The incompatibility is preserved in the offspring from the crosses

Since the incompatibility between TK and WX populations is not absolute, when incompatible males are released to suppress a target population, some hybrids will be generated. If these hybrids are compatible with the released males, then subsequent male release will help these hybrids to reproduce. This would pose a danger of gradually replacing the target population with a compatible hybrid population or creating a balance between the original target population and the hybrid population. In these cases, the populations are not effectively suppressed by male release. To test if the use of naturally incompatible populations as a control strategy is sustainable, the compatibility between the hybrids and TK and WX populations were measured.

**Figure 6:**
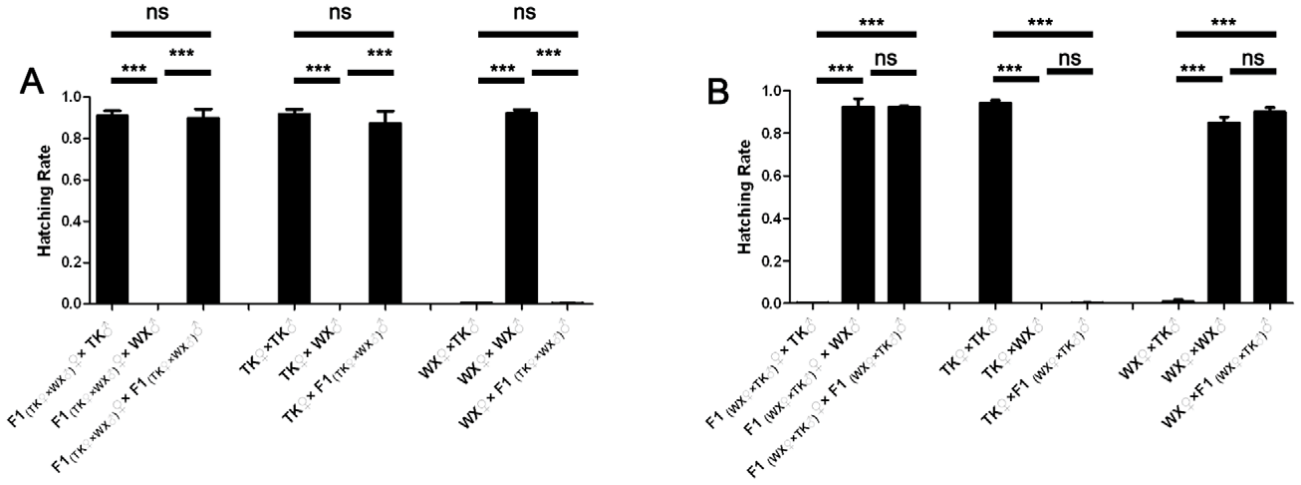
Reproductive compatibilities of F1 offspring from incompatible crosses. F1 offspring from TK and WX crosses were mixed with TK, WX or the F1. The hatching rates were compared. (A) F1 from TK♀×WX♂ cross; (B) F1 from WX♀×TK♂ cross. Error bars represent standard errors; ns, non-significant *P*-value; *** *P*<0.0001 by *t*-test.

In the case of releasing WX♂ to suppress TK population, both male and female F1 offspring will be generated from TK♀×WX♂ cross, designated as F1♀_(TK♀×WX♂)_ and F1♂_(TK♀×WX♂)_. These hybrids will encounter TK♀, TK♂ and WX♂, resulting in six possible mating combinations. Three extra mating combinations WX♀×F1♂_(TK♀×WX♂)_, WX♀×WX♂ and WX♀×TK♂ that would not accompany this population suppression measure were also included in this set to provide more information about the crossing type of F1♂_(TK♀×WX♂)_. These nine mating combinations were compared for fecundity. As shown in Table S5 and Figure 6A, the mating combinations F1♀_(TK♀×WX♂)_×F1♂_(TK♀×WX♂)_ and F1♀_(TK♀×WX♂)_×TK♂ had comparably high hatching rates, while F1♀_(TK♀×WX♂)_×WX♂ produced no larva. These indicate F1♀_(TK♀×WX♂)_ maintained the crossing type of TK♀. On the other hand, the average hatching rates of TK♀×F1♂_(TK♀×WX♂)_ and TK♀×TK♂ were comparably high, while the hatching rates in WX♀×F1♂_(TK♀×WX♂)_ and WX♀×TK♂ crosses were comparably low, indicating that F1♂_(TK♀×WX♂)_ maintained the crossing type of TK♂. These results demonstrate that in TK♀×WX♂ cross, both male and female F1 offspring maintained their maternal crossing type.

Reciprocally, the strategy of using TK♂ to suppress WX population was tested for sustainability. To test the crossing type of F1♀_(WX♀×TK♂)_ and F1♂_(WX♀×TK♂)_ generated from the cross between WX♀ and TK♂, nine possible mating combinations were compared for hatching rate. As shown in Table S5 and Figure 6B, the average hatching rates of F1♀_(WX♀×TK♂)_×F1♂_(WX♀×TK♂)_ and F1♀_(WX♀×TK♂)_×WX♂ were comparably high, while the hatching rate was low for F1♀_(WX♀×TK♂)_×TK♂ group. These indicate F1♀_(WX♀×TK♂)_ maintained the crossing type of WX♀. On the other hand, the average hatching rates of WX♀×F1♂_(WX♀×TK♂)_ and WX♀×WX♂ were comparably high, while the average hatching rates of TK♀×WX♂ and TK♀×F1♂_(WX♀×TK♂)_ were comparably low. These indicate F1♂_(WX♀×TK♂)_ maintained the crossing type of WX♂. In WX♀×TK♂ cross, both male and female F1 offspring maintained their maternal crossing type.

Taken together, when using incompatible males to suppress a target *Cx. pipiens pallens* population, hybrid offspring may be generated if the incompatibility is not absolute. However, these hybrids maintain their maternal crossing types. This phenomenon indicates that both original target population and inadvertently generated hybrids are subject to suppression by the released incompatible males, suggesting that this control strategy is sustainable.

### The three *Cx. pipiens pallens* populations are all infected with *Wolbachia*

The crossing types observed in these *Cx. pipiens pallens* populations were inherited maternally, suggesting the possibility that *Wolbachia* was the causal factor. To detect possible infections of *Wolbachia*, PCR was carried out using total DNA extracted from WX, NJ and TK populations. The primers were selected to amplify the *wsp* gene [36], [37] and the *ank2* gene [38] according to published studies. For each population, 100% infection rate was detected (30 positive out of 30 tested mosquitoes for each population). The *wsp* sequence can distinguish supergroup A, *w*Pip strains of supergroup B and *w*CauB strains of supergroup B. The *ank2* sequence can further distinguish five phylogenetic groups (*w*Pip-I to *w*Pip-V) of *w*Pip strains [38]. PCR results show that using the generic wF-wR primer pair a fragment around 600 bp was amplified from all three natural populations of *Cx. pipiens pallens* (Figure S2A). A fragment around 500 bp was amplified from all three populations using the *w*Pip-specific wBpipF-wR primer pair. Neither wAF-wR primer pair (specific for supergroup A) nor wBcauBF-wR primer pair (specific for *w*CauB of supergroup B) generated any amplification product. These results are consistent with previous reports that most mosquito-infecting *Wolbachia* are *w*Pip strains of supergroup B. This was confirmed by subsequent sequencing analysis, which also revealed that the *wsp* genes from these three populations are identical (GenBank accession numbers JX050185 - JX050187). PCR and sequencing analysis of *ank2* gene revealed that NJ population was infected by *Wolbachia* of *w*Pip-III group (GenBank accession number JX050182), while WX and TK populations were both infected by *Wolbachia* of *w*Pip-IV group (GenBank accession numbers JX050183 and JX050184). The *ank2* genes from WX and TK populations are identical (Figure S2B and Figure S3).

### The incompatibilities between different *Cx. pipiens pallens* populations are caused by *Wolbachia*

**Figure 7:**
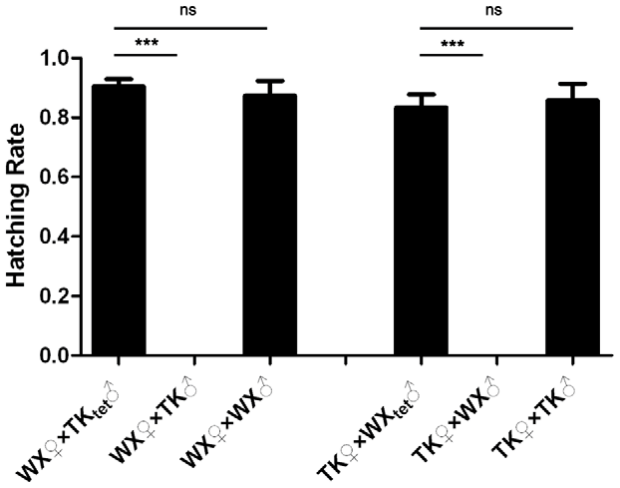
Elimination of *Wolbachia* rendered TK and WX populations fully compatible. WX females were crossed with an equal number of tetracycline-treated TK males (TK_tet_♂), untreated TK males (TK♂) or WX males (WX♂). TK females were crossed with an equal number of tetracycline-treated WX males (WX_tet_♂), untreated WX males (WX♂) or TK males (TK♂). Hatching rates were calculated for these crosses. Error bars represent standard errors; ns, non-significant *P*-value; *** *P*<0.0001 by *t*-test.

To confirm that the observed incompatibilities were caused by *Wolbachia*, the mosquitoes were treated with tetracycline [17]. The elimination of *Wolbachia* was checked by Hoechst 33342 staining [39]. As shown in Figure S4, eggs from untreated mosquitoes had strong fluorescence at both anterior and posterior ends, indicating *Wolbachia* were concentrated at these poles. In contrast, eggs from tetracycline-treated mosquitoes had even distribution of background fluorescence. No strong fluorescence was observed around the micropyle or at the posterior end. These results indicate that *Wolbachia* were removed from these mosquito populations. In addition, tetracycline-treated strains all tested negative for *Wolbachia* by PCR using *wsp*-specific primers wF and wR (data not shown). Crossing experiments were carried out using both *Wolbachia*-positive and *Wolbachia*-negative populations. WX females were crossed with *Wolbachia*-positive TK males (TK♂), *Wolbachia*-negative TK males (TK_tet_♂) and WX males. Similarly, TK females were crossed with *Wolbachia*-positive WX males (WX♂), *Wolbachia*-negative WX males (WX_tet_♂) and TK males. As shown in Figure 7, the hatching rate of WX♀×TK_tet_♂ cross was not significantly different from that of WX♀×WX♂ cross, but was significantly higher than that of WX♀×TK♂ cross. The hatching rate of TK♀×WX_tet_♂ cross was not significantly different from that of TK♀×TK♂ cross, but was significantly higher than that of TK♀×WX♂ cross. These results indicate that the bi-directional incompatibility between TK and WX populations is dependent on the presence of *Wolbachia*, i.e., it is *Wolbachia*-induced CI.

### Accession numbers

The GenBank accession numbers for sequences mentioned in the paper are ankyrin domain protein *ank2* genes of *Wolbachia* in Nanjing, Tangkou and Wuxi populations of *Cx. pipiens pallens* (JX050182 - JX050184), and surface protein precursor *wsp* genes of *Wolbachia* in Nanjing, Tangkou and Wuxi populations of *Cx. pipiens pallens* (JX050185 - JX050187).

## Discussion

Biological control is a low-pollution component of integrated pest management. A variety of strategies have been developed to suppress populations of insect pests. Taking advantage of many insects' monogamous mating behavior, SIT uses sterile male insects to compete with normal males for their mates, which results in some females producing no or a reduced number of offspring. Two common methods to produce sterile male insects are irradiation and chemical treatments. SIT has been reported to be successful in a number of trials [13], [40]–[42]. One potential problem with SIT is the dosage of radiation or sterilizing agent used to treat male insects. Insufficient dosages might result in the release of fertile males while overtreatment might damage the males so much that they cannot compete with normal male insects. In addition, the residual chemicals in treated insects could potentially cause pollution to the environment and harm untargeted insect species [43], [44].

Alternatively, IIT exploits incompatibilities between insect populations and uses incompatible male insects to compete with compatible insects for their mates. Because IIT relies on the genetic traits of incompatible populations, it could be more reproducible than SIT. Most IITs are based on *Wolbachia*-induced cytoplasmic incompatibility. Infection with *Wolbachia* has been reported in many species, including some disease vectors such as *Aedes albopictus* and *Culex quinquefasciatus* ( = *Cx. pipiens fatigans*) [15], [21], [30]. For naturally uninfected insects, artificial *Wolbachia* infection would generate an incompatible strain that could be used to suppress target populations [28]. Introgression using *Wolbachia*-infected females and *Wolbachia*-negative target population males can also be used to generate an incompatible strain. For *Wolbachia*-positive target populations, a common approach is introgression with females from another strain infected with an incompatible *Wolbachia*. Sometimes, antibiotic treatment to eliminate *Wolbachia* from target strain is needed to facilitate the introgression process [29]. The advantages of this strategy is the generation of insect population with a desired host genetic background (often to match that of the target population) to increase the likelihood of survival in the target environment and successful competition with native males. However, both artificial infection and introgression can be technically difficult. Even with successful establishment of an incompatible strain, because of the placement of *Wolbachia* in a different host genetic background, the long-term stability of this new symbiosis needs to be tested.

In our study, we exploited incompatibilities between naturally existent populations from different geographic locations to suppress mosquito populations. This spares the time and effort to create incompatible populations. Another advantage is that the stability of *Wolbachia* can be assured since this symbiotic relationship has been naturally selected for a long time. IIT involves repeated release of incompatible males that can competitively inseminate females in a target population. Since these males are not meant to have offspring, any potential adverse effect of their genetic makeup on their fitness in the target environment would not be an issue so long as these males can live long enough to mate and are sexually competitive. We tested the mating behavior of three populations collected from different locations. When mixed with males of a different population, females became inseminated, as spermatozoa were present in the spermathecal capsules of these females. TK females mixed with NJ males or WX males laid comparable number of egg rafts, although these eggs had much lower hatching rates. NJ females or WX females also laid comparable numbers of egg rafts when mixed with TK males compared to when they were mixed with males of their own populations. Egg rafts with low hatching rate also showed clear embryonic development. All these data indicate that these males and females from different populations could successfully mate. Geographical and chronic isolation did not appear to create a mating barrier between these populations.

IIT works effectively only if the incompatible males are competitive enough. Although these mosquito populations could mate with each other, we also tested if the females had strong preference of their cognate males in the presence of both cognate and incompatible males using a mating competition experiment. Our results indicated significant mating preference was not observed. In Figure 5, a linear curve would indicate random mating, while a strong preference of cognate males would result in an arch curve with the median value greater than the average of the two extrema. Both curves are basically linear, supporting that these females chose their mates randomly. With an increasing ratio of incompatible to cognate males, the number of offspring decreased. Our results are consistent with previous reports from other groups that assortative mating usually does not occur in natural populations when numerous *Wolbachia* strains coexist within those populations [45]. These data suggest that although *Wolbachia* may cause CI in mosquitoes and skew survival in favor of those embryos that harbor the same or compatible *Wolbachia*, it does not affect the mate selection significantly. Instead, insects including mosquitoes select their mates based on their own genetic traits [46], [47]. The selection of suitable populations for mosquito control would depend on finding a population whose genome is compatible with that of target population, while also having a *Wolbachia* infection that induces cytoplasmic incompatibility in that context.

Another factor that influences IIT success is the monogamous mating behavior of females. After female mosquitoes are inseminated, they usually become refractory to re-mating. This phenomenon has been attributed to the effects of proteins in the seminal fluid received from male mosquitoes. But female monogamy is not always absolute. Polyandry has been reported in a number of mosquito species, such as *Aedes aegypti* and *Culex tarsalis*. The likelihood of female re-mating increases after these females go through gonotrophic cycles [48], [49], possibly due to the waning of seminal fluid proteins. One potential obstacle to the use of mosquitoes from different geographic locations to suppress target populations is the incompatibility between ligands in seminal fluid of one population and receptors in females of the other. In this scenario, insemination would not prevent the female mosquitoes from mating with reproductively compatible males. In our study, TK female mosquitoes became refractory to re-mating after being inseminated by WX or NJ male mosquitoes, even after these females had gone through a gonotrophic cycle, indicating seminal fluid proteins of WX or NJ population can act on receptors of TK population to cause post-coital behavioral changes in TK females. These results suggest that the seminal fluid proteins and their receptors are conserved enough between these populations, or their interactions are flexible enough to tolerate certain mutations. Further studies are needed to reveal the interactions between seminal fluid proteins and their receptors of different mosquito populations. Potentially, there are other factors causing female monogamy. If so, these factors seem to be functional across different populations.

Population suppression usually requires repeated effort. It is important for IIT to work for many generations. We tested if hybrids of incompatible populations became compatible with their parental populations. Our data indicate the incompatibility between different mosquito populations is hereditarily stable. This suggests that using males from incompatible natural populations to suppress a target population is sustainable. Although in our study, F1 offspring from incompatible crosses maintained the maternal crossing types, it should be noted that the preservation of maternal crossing type is not always the case. For example, F1♀ from the cross of bi-directionally incompatible *Cx. quinquefasciatus* strains Bei and Pel became fully compatible with Pel males [50]. This cautions us that when choosing incompatible populations, the crossing types of F1 hybrids should be carefully tested as well.

Although the climates at the sites where these mosquitoes originated from are different due to their latitudes, etc., these mosquitoes did not demonstrate significant behavioral differences in the same artificial environment of our insectary. More trials are needed to determine how they would behave and interact in semi-field and field trials. It remains to be tested whether incompatible male mosquitoes released into different natural environments will remain sexually competitive, and reduce the fecundity of local females.

Both maternal inheritance of crossing types and elimination of incompatibility by tetracycline treatment indicate that the observed incompatibility is caused by *Wolbachia*. To test if these mosquito populations are infected with different strains of *Wolbachia*, specific DNA fragments were PCR amplified from three mosquito populations. We sequenced *wsp* and *ank2* genes, two specific genes commonly used to type *Wolbachia*. However, sequence analysis shows that *wsp* gene is identical in these three mosquito populations. The *ank2* gene is identical between TK and WX populations, while *ank2* gene of NJ population is different from TK or WX population. These *Wolbachia* are all *w*Pip strains of supergroup B. It is currently unclear how they differ from each other. The definitive answer would require more sequencing or other typing tests which are beyond the scope of the current study. ANK genes (including *ank2*) were initially proposed to be involved in CI [50]. This hypothesis was challenged by subsequent analysis that failed to find any association of ANK polymorphism and CI [38]. Our data show NJ and WX populations are bi-directionally compatible, yet their *ank2* genes are different. On the other hand, TK and WX populations are bi-directionally incompatible, yet their *ank2* genes are identical (or at least the sequenced segments). These provide support to the view that homology between these typing markers does not correlate to the level of CI, suggesting molecular markers that are polymorphic and more closely associated with CI factors are yet to be found [38], [51]. Nuclear contribution to incompatibility has also been reported [50]. Being isolated from each other in the wild, these mosquitoes might have accumulated enough mutations in their genomes and/or even mitochondrial DNA to make some crosses incompatible. These differences in the host could directly cause incompatibility or more likely cause incompatibility by modulating *Wolbachia* gene expression. Consequently, although the incompatibilities between these populations are dependent on *Wolbachia*, contribution of host genomes cannot be ruled out at present. Our observation of embryonic development of incompatible eggs is in accordance with previously reported *Wolbachia*-mediated CI. It has been reported that incompatible eggs from two *Wolbachia*-positive populations have higher level of development than incompatible eggs from *Wolbachia*-negative females (which are usually undistinguishable from unfertilized eggs) [34]. We observed stemmata, bristles and segmentation in both TK♀×WX♂ and TK♀×NJ♂ crosses. This would be consistent with the multi-factorial *mod resc* model: even incompatible *Wolbachia* provides partial rescue in the eggs [15].

Although incompatibility between insect populations (including CI) is not fully understood, its applicability is promising. Our study proves that IIT is a feasible control strategy for *Cx. pipiens pallens*. The use of naturally occurring populations without genetic manipulation will save time and effort, and require less technical knowhow. This is an advantage for many less developed regions that deserves consideration. The wide distribution of mosquitoes in varied environments may be turned against them because it provides rich diversity in incompatibility; so that it is likely to find naturally incompatible and sexually competitive strains for many target populations. The conclusions from our study on *Cx. pipiens pallens* might offer reference to control measures of other mosquitoes, as well.

## Supporting Information

Figure S1 **Embryonic development of eggs from incompatible crosses.** Eggs from incompatible crosses TK♀×WX♂ and TK♀×NJ♂ were compared to unfertilized eggs and eggs from compatible cross TK♀×TK♂ using microscopy. (A) unfertilized egg of TK♀ 48 hours after oviposition, (B) egg from TK♀×NJ♂ 48 hours after oviposition, (C) egg from TK♀×WX♂ 48 hours after oviposition, (D) egg from compatible cross (TK♀×TK♂) 36 hours after oviposition, (E) Egg from TK♀×TK♂ 48 hours after oviposition. Both TK♀×WX♂ and TK♀×NJ♂ eggs displayed signs of embryonic development. In (E), a first-instar larva already released into water, leaving behind an empty eggshell with an opened operculum.
(TIF)

Figure S2 **Detection of** ***Wolbachia*** **in NJ, WX and TK populations.** (A) PCR amplification of *Wolbachia wsp* gene. Four PCR reactions were performed in parallel for each DNA specimen (Lanes 1, 4, 7, 10 for TK population; Lanes 2, 5, 8, 11 for NJ population; Lanes 3, 6, 9, 12 for WX population). Lanes 1–3 were amplified with primers wF and wR, Lanes 4–6 were amplified with primers wAF and wR, Lanes 7–9 were amplified with primers wBpipF and wR, Lanes 10–12 were amplified with primers wBcauBF and wR. (B) PCR amplification of *Wolbachia ank2* gene. Lane 1: TK population, Lane 2: NJ population, Lane 3: WX population.
(TIF)

Figure S3 **Alignment of** ***ank2*** **sequences from NJ, WX and TK populations.** Alignment of amplified fragments of *Wolbachia ank2* genes from NJ, WX and TK populations with two published sequences *ank2-c* (GenBank accession number AM397070.1) and *ank2-d* (GenBank accession number AM397071.1). NJ sequence shares 100% homology with *ank2-c*, WX and TK sequences are identical and share 100% homology with *ank2-d*.
(TIF)

Figure S4 **Elimination of** ***Wolbachia*** **from TK, WX and NJ populations by tetracycline treatment.** Eggs of tetracycline-treated and untreated mosquitoes were stained with Hoechst 33342 and visualized by fluorescence microscopy. (A) *Wolbachia* are concentrated at both anterior and posterior ends of untreated eggs indicated by strong fluorescence. (B) The same egg as in (A) under visible light. (C) Tetracycline-treated egg shows no *Wolbachia* distribution at either end of the egg. (D) The same egg as in (C) under visible light. Shown here are results of TK population. Treatment of WX and NJ populations had similar results. Scale bar, 0.1 mm.
(TIF)

Table S1 **Reciprocal crosses among** ***Cx. pipiens pallens*** **field populations.**
(PDF)

Table S2 **Second round mating combinations of females retrieved from incompatible crosses.**
(PDF)

Table S3 **Mating combinations of TK and WX females with both cognate and an increasing number of incompatible males.**
(PDF)

Table S4 **Second round mating combinations of females retrieved from mixed male populations.**
(PDF)

Table S5 **Mating combinations of F1 offspring of TK and WX crosses.**
(PDF)

Table S6 **Crosses of tetracycline-treated and untreated WX and TK populations.**
(PDF)
