# Supplementary figures and images for: Naturally Occurring Incompatibilities between Different Culex pipiens pallens Populations as the Basis of Potential Mosquito Control Measures
Source: PLoS Negl Trop Dis. 2013 Jan 31;7(1):e2030. doi: 10.1371/journal.pntd.0002030 (PMC3561155; doi:10.1371/journal.pntd.0002030)

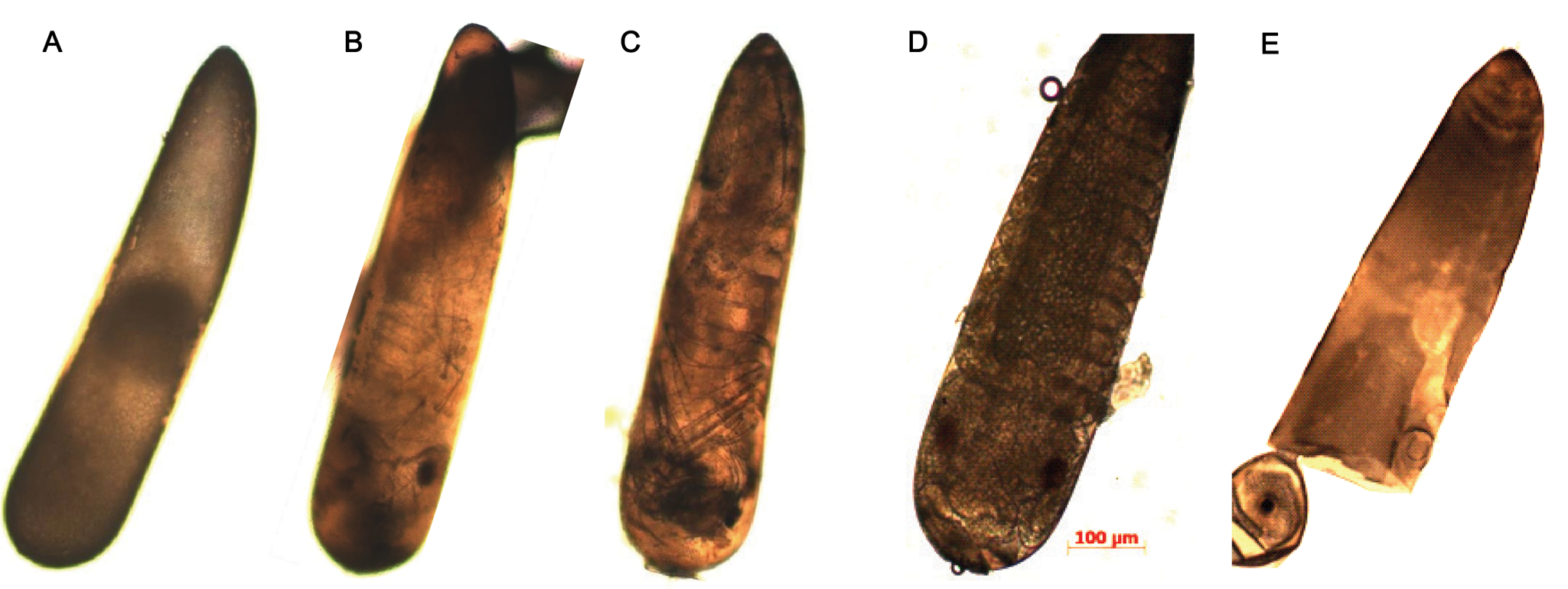

Supplement: Figure S1 — Embryonic development of eggs from incompatible crosses. Eggs from incompatible crosses TK♀×WX♂ and TK♀×NJ♂ were compared to unfertilized eggs and eggs from compatible cross TK♀×TK♂ using microscopy. (A) unfertilized egg of TK♀ 48 hours after oviposition, (B) egg from TK♀×NJ♂ 48 hours after oviposition, (C) egg from TK♀×WX♂ 48 hours after oviposition, (D) egg from compatible cross (TK♀×TK♂) 36 hours after oviposition, (E) Egg from TK♀×TK♂ 48 hours after oviposition. Both TK♀×WX♂ and TK♀×NJ♂ eggs displayed signs of embryonic development. In (E), a first-instar larva already released into water, leaving behind an empty eggshell with an opened operculum. (TIF) [file pntd.0002030.s001.tif]

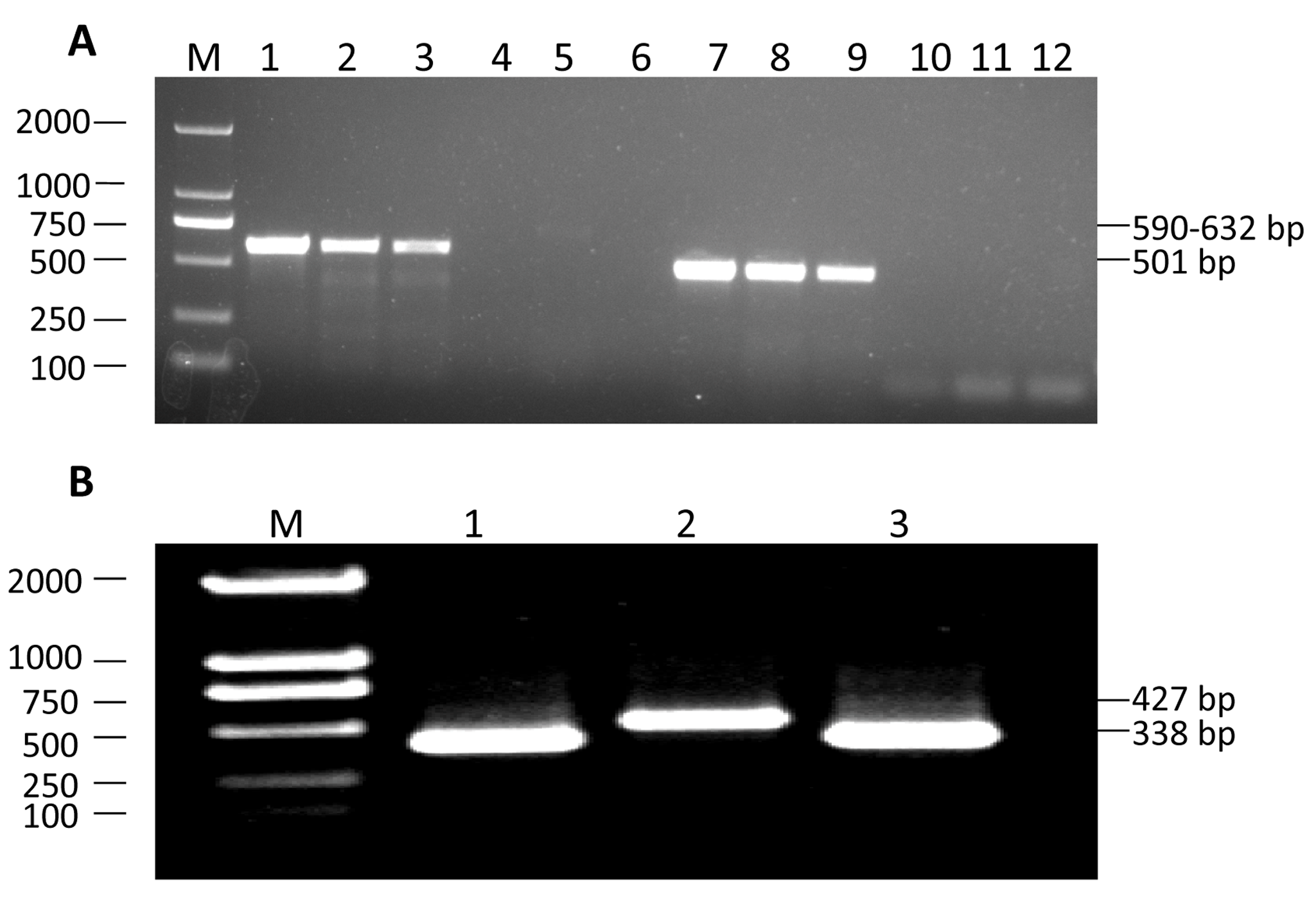

Supplement: Figure S2 — Detection of Wolbachia in NJ, WX and TK populations. (A) PCR amplification of Wolbachia wsp gene. Four PCR reactions were performed in parallel for each DNA specimen (Lanes 1, 4, 7, 10 for TK population; Lanes 2, 5, 8, 11 for NJ population; Lanes 3, 6, 9, 12 for WX population). Lanes 1–3 were amplified with primers wF and wR, Lanes 4–6 were amplified with primers wAF and wR, Lanes 7–9 were amplified with primers wBpipF and wR, Lanes 10–12 were amplified with primers wBcauBF and wR. (B) PCR amplification of Wolbachia ank2 gene. Lane 1: TK population, Lane 2: NJ population, Lane 3: WX population. (TIF) [file pntd.0002030.s002.tif]

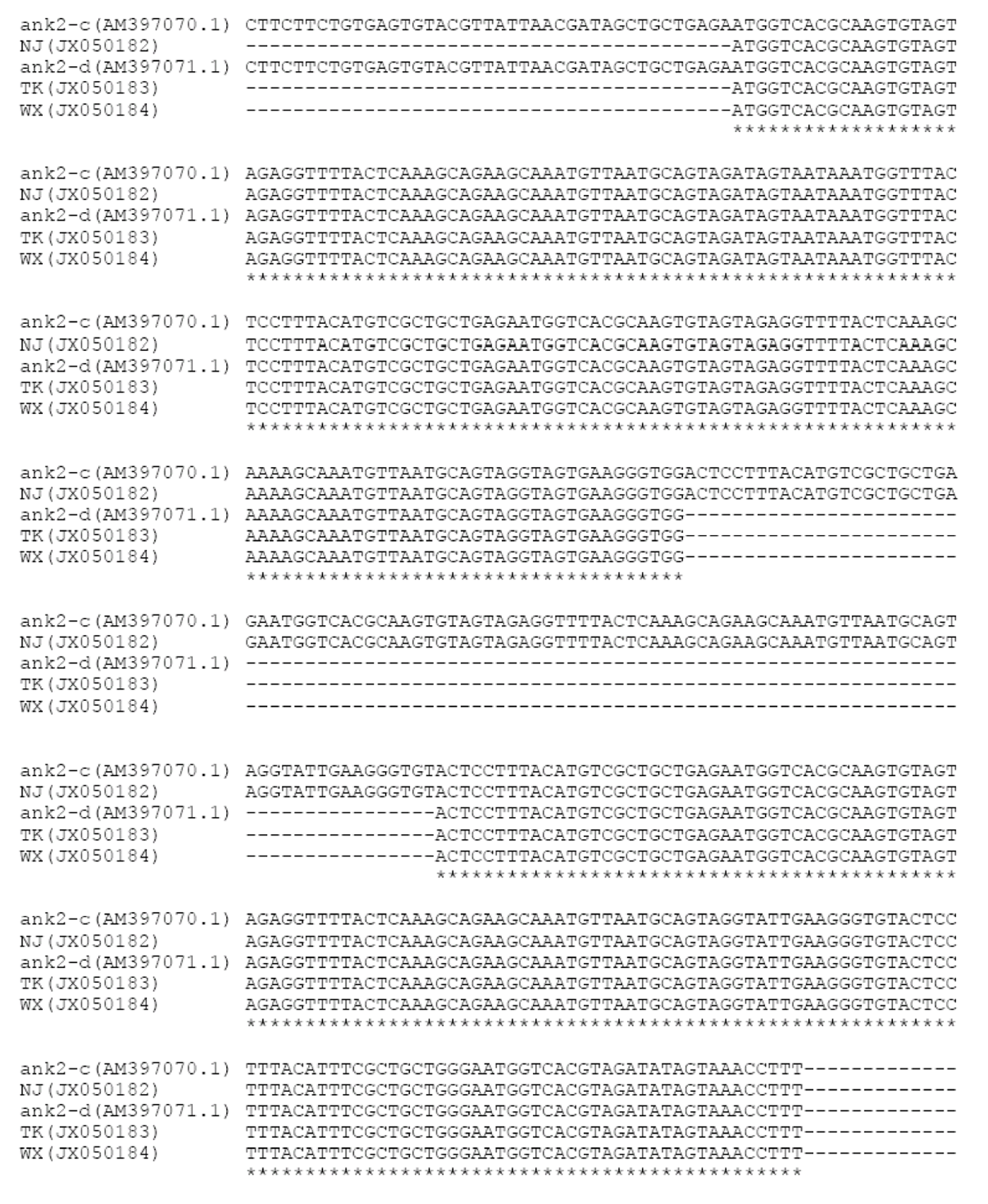

Supplement: Figure S3 — Alignment of ank2 sequences from NJ, WX and TK populations. Alignment of amplified fragments of Wolbachia ank2 genes from NJ, WX and TK populations with two published sequences ank2-c (GenBank accession number AM397070.1) and ank2-d (GenBank accession number AM397071.1). NJ sequence shares 100% homology with ank2-c, WX and TK sequences are identical and share 100% homology with ank2-d. (TIF) [file pntd.0002030.s003.tif]

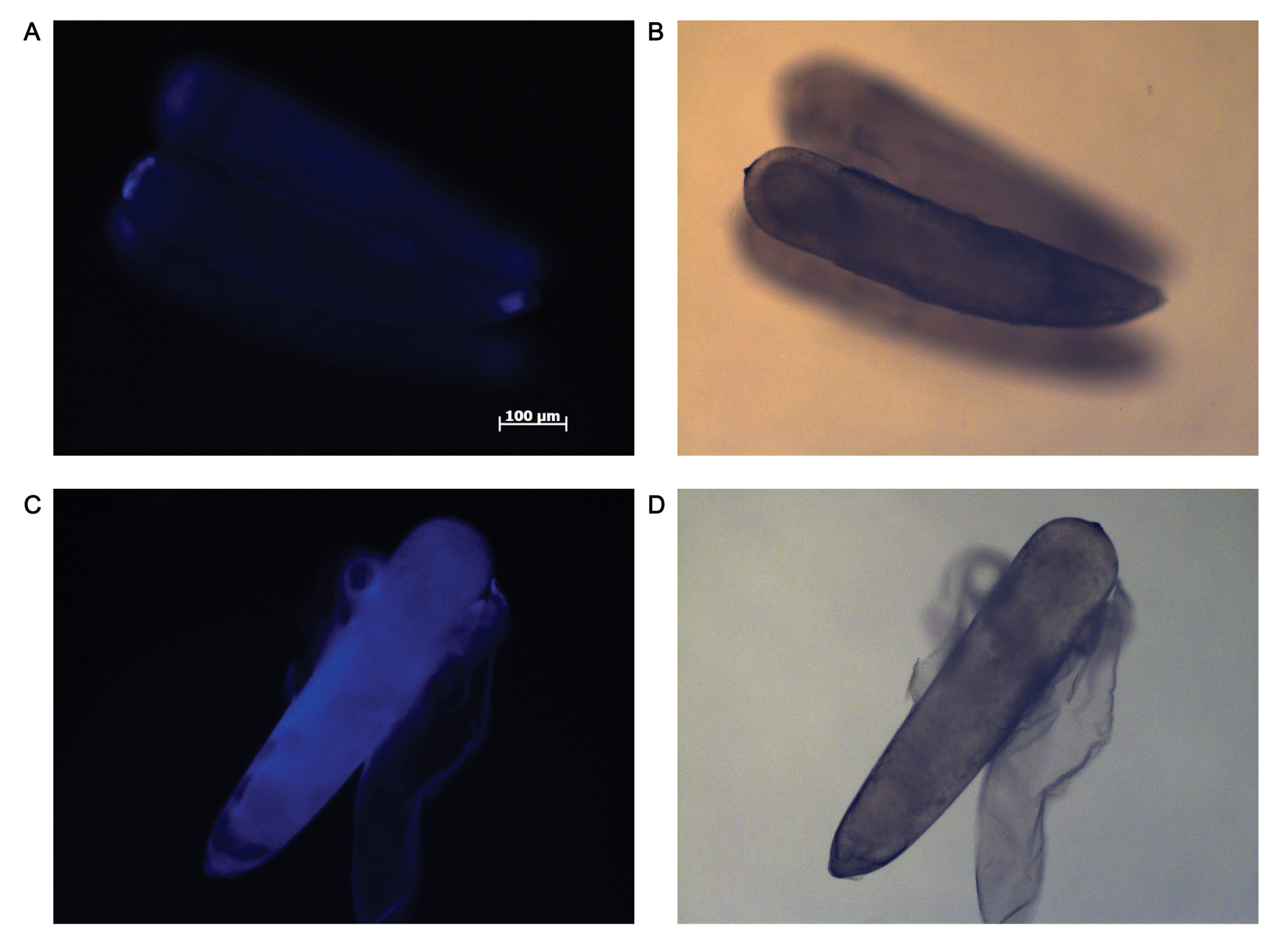

Supplement: Figure S4 — Elimination of Wolbachia from TK, WX and NJ populations by tetracycline treatment. Eggs of tetracycline-treated and untreated mosquitoes were stained with Hoechst 33342 and visualized by fluorescence microscopy. (A) Wolbachia are concentrated at both anterior and posterior ends of untreated eggs indicated by strong fluorescence. (B) The same egg as in (A) under visible light. (C) Tetracycline-treated egg shows no Wolbachia distribution at either end of the egg. (D) The same egg as in (C) under visible light. Shown here are results of TK population. Treatment of WX and NJ populations had similar results. Scale bar, 0.1 mm. (TIF) [file pntd.0002030.s004.tif]
