# Supplementary material for: Naturally Occurring Incompatibilities between Different Culex pipiens pallens Populations as the Basis of Potential Mosquito Control Measures
Source: PLoS Negl Trop Dis. 2013 Jan 31;7(1):e2030. doi: 10.1371/journal.pntd.0002030 (PMC3561155; doi:10.1371/journal.pntd.0002030)
Supplement: Table S1 — Reciprocal crosses among Cx. pipiens pallens field populations. (PDF) [file pntd.0002030.s005.pdf]

Table S1 Reciprocal crosses among *Cx. pipiens pallens* field populations

Cross	Mating Combination*	Total Egg Rafts	Total Eggs	Total Larvae	Hatching Rate	Comparison	Significance
A	TK♀(29)×TK♂(29)	22	2788	2433	0.873±0.028	A *vs.* B	*P*<0.0001(t=28.219;df=38)
B	TK♀(29)×WX♂(29)	18	2194	2	0.001±0.001		
C	WX♀(25)×WX♂(25)	18	2069	1799	0.856±0.003	C *vs.* D	*P*<0.0001(t=34.463;df=37)
D	WX♀(25)×TK♂(25)	21	2384	14	0.005±0.026		
E	TK♀(33)×TK♂(33)	25	3419	3087	0.883±0.028	E *vs.* F	*P*<0.0001(t=30.290;df=47)
F	TK♀(33)×NJ♂(33)	24	3148	12	0.004±0.003		
G	NJ♀(32)×TK♂(32)	31	3452	13	0.004±0.002	G *vs.* H	*P*<0.0001(t=-63.584;df=58)
H	NJ♀(32)×NJ♂(32)	29	3748	3474	0.922±0.015		
I	NJ♀(33)× NJ♂(33)	31	3896	3470	0.882±0.019	I *vs.* J	NS(t=-0.724;df=59;*P*=0.472)
J	NJ♀(33)×WX♂(33)	30	3954	3577	0.898±0.011		
K	WX♀(32)×NJ♂(32)	23	3178	2664	0.831±0.034	K *vs.* L	NS(t=-0.570;df=44;*P*=0.571)
L	WX♀(32)×WX♂(32)	23	3509	3072	0.859±0.036		

* Numbers in parentheses refer to the numbers of mosquitoes used in the respective combinations.

For each cross, hatching rate value is expressed as mean± standard error. NS, nonsignificant *P*-value.
