# Supplementary material for: Naturally Occurring Incompatibilities between Different Culex pipiens pallens Populations as the Basis of Potential Mosquito Control Measures
Source: PLoS Negl Trop Dis. 2013 Jan 31;7(1):e2030. doi: 10.1371/journal.pntd.0002030 (PMC3561155; doi:10.1371/journal.pntd.0002030)
Supplement: Table S2 — Second round mating combinations of females retrieved from incompatible crosses. (PDF) [file pntd.0002030.s006.pdf]

Table S2 Second round mating combinations of females retrieved from incompatible crosses

Cross	Mating combination*	Total egg rafts	Total Eggs	Total larvae	Hatching Rate	Comparison	Significance
1	♀$_{\text{retrieved from (TK♀×WX♂)}}$ (13) × TK ♂ (13)	10	1022	1	0.001±0.001	1 *vs.* 2	NS(t=-1.013,df=18,*P*=0.324)
2	♀$_{\text{retrieved from (TK♀×WX♂)}}$ (13) × No ♂ (0)	10	1101	5	0.005±0.004		
3	♀$_{\text{retrieved from(WX♀×TK♂)}}$ (9) × WX ♂ (9)	4	388	0	0.000±0.000	3 *vs.* 4	NS(t=-1.0,df=6,*P*=0.356)
4	♀$_{\text{retrieved from(WX♀×TK♂)}}$ (9) × No ♂ (0)	4	372	1	0.003±0.003		

* Numbers in parentheses refer to the numbers of mosquitoes used in the respective combinations.

For each cross, hatching rate value is expressed as mean± standard error. NS, nonsignificant *P*-value.
