# Supplementary material for: Naturally Occurring Incompatibilities between Different Culex pipiens pallens Populations as the Basis of Potential Mosquito Control Measures
Source: PLoS Negl Trop Dis. 2013 Jan 31;7(1):e2030. doi: 10.1371/journal.pntd.0002030 (PMC3561155; doi:10.1371/journal.pntd.0002030)
Supplement: Table S3 — Mating combinations of TK and WX females with both cognate and an increasing number of incompatible males. (PDF) [file pntd.0002030.s007.pdf]

Table S3 Mating combinations of TK and WX females with both cognate and increasing number of incompatible males

| Cross | Mating combination* | Total egg rafts | Total Eggs | Total larvae | Hatching Rate | Comparison | Significance |
|---|---|---|---|---|---|---|---|
| M | TK♀(31) ×No♂(0) | 5 | 560 | 0 | 0.000±0.000 | | |
| N | TK♀(31) ×TK♂(31) | 26 | 3753 | 3304 | 0.861±0.020 | | |
| O | TK♀(31) ×[TK♂(31) + WX♂(31)] | 31 | 4830 | 830 | 0.211±0.071 | O *vs.* N | *P*<0.0001(t=-8.135 df=55) |
| P | TK♀(31) ×[TK♂(31) + 3xWX♂(93)] | 30 | 4521 | 198 | 0.063±0.039 | P *vs.* N | *P*<0.0001(t=-17.483 df=54) |
| Q | TK♀(31) ×WX♂(31) | 30 | 4668 | 19 | 0.004±0.003 | Q *vs.* N | *P*<0.0001(t=-44.891 df=54) |
| | | | | | | | |
| R | WX♀(46) × No♂(0) | 8 | 920 | 0 | 0.000±0.000 | | |
| S | WX♀(46) × WX♂(46) | 37 | 5061 | 4616 | 0.903±0.013 | | |
| T | WX♀(46) × [WX♂(46) +TK♂(46)] | 43 | 5521 | 3341 | 0.607±0.066 | T *vs.* S | *P*<0.0001(t=-4.142 df=78) |
| U | WX♀(46) × [WX♂(46) + 3xTK♂(138)] | 36 | 5242 | 2256 | 0.407±0.077 | U *vs.* S | *P*<0.0001(t=-6.49 df=71) |
| V | WX♀(46) × TK♂(46) | 39 | 5927 | 9 | 0.001±0.001 | V *vs.* S | *P*<0.0001(t=-73.844 df=74) |

* Numbers in parentheses refer to the numbers of mosquitoes used in the respective combinations.

For each cross, hatching rate value is expressed as mean± standard error. NS, nonsignificant *P*-value.
