# Supplementary material for: Naturally Occurring Incompatibilities between Different Culex pipiens pallens Populations as the Basis of Potential Mosquito Control Measures
Source: PLoS Negl Trop Dis. 2013 Jan 31;7(1):e2030. doi: 10.1371/journal.pntd.0002030 (PMC3561155; doi:10.1371/journal.pntd.0002030)
Supplement: Table S4 — Second round mating combinations of females retrieved from mixed male populations. (PDF) [file pntd.0002030.s008.pdf]

Table S4 Second round mating combinations of females retrieved from mixed male populations

Cross	Mating combination*	Total egg rafts	Total Eggs	Total larvae	Hatching Rate	Comparison	Significance
9	♀ retrieved from (TK♀×No♂) (11) ×TK ♂ (11)	11	1371	873	0.630±0.113	9 *vs.* 10	*P*<0.05 (t=2.289 df=11 *P*=0.043)
10	♀ retrieved from (TK♀×No♂) (11) ×No ♂ (0)	2	194	0	0.000±0.000		
11	♀ retrieved from (TK♀×TK♂) (11) ×TK ♂ (11)	7	832	736	0.860±0.038	11 *vs.* 12	NS(t=-0.694 df=15 *P*=0.498)
12	♀ retrieved from (TK♀×TK♂) (11) ×No ♂ (0)	10	1054	936	0.884±0.011		
13	♀ retrieved from [TK♀×(TK♂+ WX♂)] (14) ×TK ♂ (14)	13	1633	293	0.190±0.101	13 *vs.* 14	NS(t=0.407 df=24 *P*=0.687)
14	♀ retrieved from [TK♀×(TK♂+ WX♂)] (14) ×No ♂ (0)	13	1701	212	0.135±0.090		
15	♀ retrieved from[TK♀×(TK♂+3xWX♂)] (13) ×TK ♂ (13)	13	1758	1	0.0004±0.0004	15 *vs.* 16	NS(t=-1.142 df=21 *P*=0.266)
16	♀ retrieved from[TK♀×(TK♂+3xWX♂)] (13) ×No ♂ (0)	10	1298	93	0.081±0.081		
17	♀ retrieved from (TK♀×WX♂) (14) ×TK ♂ (14)	9	1137	6	0.006±0.006	17 *vs.* 18	NS(=0.882 df=21 *P*=0.388)
18	♀ retrieved from (TK♀×WX♂) (14) ×No ♂ (0)	14	1595	2	0.002±0.001		
19	♀ retrieved from(WX♀×No♂) (20) ×WX ♂ (20)	7	857	361	0.431±0.157	19 *vs.* 20	*P*<0.05 (t=2.750 df=12 *P*=0.018)
20	♀ retrieved from(WX♀×No♂) (20)×No ♂ (0)	7	782	0	0.000±0.000		
21	♀ retrieved from(WX♀×WX♂) (21)×WX ♂ (21)	16	1969	1630	0.828±0.025	21 *vs.* 22	NS(t=-0.083 df=30 *P*=0.934)
22	♀ retrieved from(WX♀×WX♂) (21) ×No ♂ (0)	16	1806	1527	0.823±0.043		
23	♀ retrieved from[WX♀×(WX♂+TK♂)] (22)×WX ♂ (22)	19	2077	1136	0.493±0.093	23 *vs.* 24	NS(t=-0.369 df=33 *P*=0.714)
24	♀ retrieved from[WX♀×(WX♂+TK♂)] (22)×No ♂ (0)	16	1975	1085	0.544±0.105		
25	♀ retrieved from[WX♀×(WX♂+3xTK♂)] (21)×WX ♂ (21)	18	2120	699	0.355±0.096	25 *vs.* 26	NS(t=-0.357 df=30 *P*=0.724)
26	♀ retrieved from[WX♀×(WX♂+3xTK♂)] (21)×No ♂ (0)	14	1771	679	0.408±0.112		
27	♀ retrieved from(WX♀×TK♂) (21)×WX ♂ (21)	19	2304	20	0.007±0.006	27 *vs.* 28	NS(t=0.806 df=33 *P*=0.426)
28	♀ retrieved from(WX♀×TK♂) (21)×No ♂ (0)	16	2078	9	0.002±0.002		

* Numbers in parentheses refer to the numbers of mosquitoes used in the respective combinations.

For each cross, hatching rate value is expressed as mean± standard error. NS, nonsignificant *P*-value.
