# Supplementary material for: Naturally Occurring Incompatibilities between Different Culex pipiens pallens Populations as the Basis of Potential Mosquito Control Measures
Source: PLoS Negl Trop Dis. 2013 Jan 31;7(1):e2030. doi: 10.1371/journal.pntd.0002030 (PMC3561155; doi:10.1371/journal.pntd.0002030)
Supplement: Table S5 — Mating combinations of F1 offspring of TK and WX crosses. (PDF) [file pntd.0002030.s009.pdf]

Table S5 Mating combinations of F1 offspring of TK and WX crosses

Cross	Mating combination*	Total egg rafts	Total Eggs	Total larvae	Hatching Rate	Comparison	Significance
D1	$F1_{(WX♀×TK♂)}$♀(7)× TK♂(7)	7	1072	1	0.001±0.001	D1 *vs.* D2	*P*<0.0001(t=-28.098 df=10)
D2	$F1_{(WX♀×TK♂)}$♀(7) × WX♂(7)	5	900	835	0.922±0.040	D2 *vs.* D3	NS (t=0.055 df=8 *P*=0.958)
D3	$F1_{(WX♀×TK♂)}$♀(7)× $F1_{(WX♀×TK♂)}$♂(7)	5	779	716	0.919±0.007	D1 *vs.* D3	*P*<0.0001(t=-165.351 df=10)
D4	TK♀(10) × TK♂(10)	7	1091	1029	0.940±0.014	D4 *vs.* D5	*P*<0.0001(t=68.104 df=12)
D5	TK♀(10) × WX♂(10)	7	1046	0	0.000±0.000	D5 *vs.* D6	NS (t=-0.875 df=14 *P*=0.396)
D6	TK♀(10) × $F1_{(WX♀×TK♂)}$♂(10)	9	1558	3	0.002±0.002	D4 *vs.* D6	*P*<0.0001(t=76.332 df=14)
D7	WX♀(10)× TK♂(10)	7	941	10	0.009±0.009	D7 *vs.* D8	*P*<0.0001(t=-27.856 df=13)
D8	WX♀(10)× WX♂(10)	8	1207	917	0.847±0.027	D8 *vs.* D9	NS (t=-1.382 df=12 *P*=0.192)
D9	WX♀(10)× $F1_{(WX♀×TK♂)}$♂(10)	6	851	763	0.898±0.022	D7 *vs.* D9	*P*<0.0001(t=-39.975 df=11)
B1	$F1_{(TK♀×WX♂)}$♀(9)× TK♂(9)	7	1314	1194	0.908±0.025	B1 *vs.* B2	*P*<0.0001(t=42.365 df=14)
B2	$F1_{(TK♀×WX♂)}$♀(9)× WX♂(9)	9	1558	0	0.000±0.000	B1 *vs.* B3	NS(t=0.2 df=13 *P*=0.845)
B3	$F1_{(TK♀×WX♂)}$♀(9)× $F1_{(TK♀×WX♂)}$♂(9)	8	1453	1316	0.897±0.126	B2 *vs.* B3	*P*<0.0001(t=-21.417 df=15)
B4	TK♀(9)× TK♂(9)	6	793	739	0.916±0.026	B4 *vs.* B5	*P*<0.0001(t=38.000 df=11)
B5	TK♀(9)× WX♂(9)	7	888	0	0.000±0.000	B4 *vs.* B6	NS(t=0.713 df=9 *P*=0.494)
B6	TK♀(9)× $F1_{(TK♀×WX♂)}$♂(9)	5	818	712	0.872±0.060	B5 *vs.* B6	*P*<0.0001(t=-17.425 df=10)
B7	WX♀(9)× TK♂(9)	7	970	2	0.002±0.001	B7 *vs.* B8	*P*<0.0001(t=-57.272 df=11)
B8	WX♀(9)× WX♂(9)	6	864	793	0.921±0.017	B8 *vs.* B9	*P*<0.0001(t=52.845 df=10)
B9	WX♀(9)× $F1_{(TK♀×WX♂)}$♂(9)	6	622	1	0.001±0.001	B7 *vs.* B9	NS(t=0.636 df=11 *P*=0.538)

* Numbers in parentheses refer to the numbers of mosquitoes used in the respective combinations.

For each cross, hatching rate value is expressed as mean± standard error. NS, nonsignificant *P*-value.
