# Supplementary material for: Naturally Occurring Incompatibilities between Different Culex pipiens pallens Populations as the Basis of Potential Mosquito Control Measures
Source: PLoS Negl Trop Dis. 2013 Jan 31;7(1):e2030. doi: 10.1371/journal.pntd.0002030 (PMC3561155; doi:10.1371/journal.pntd.0002030)
Supplement: Table S6 — Crosses of tetracycline-treated and untreated WX and TK populations. (PDF) [file pntd.0002030.s010.pdf]

Table S6 Crosses of tetracycline–treated and untreated WX and TK populations

Cross	Mating Combination*	Total Egg Rafts	Total Eggs	Total Larvae	Hatching Rate	Comparison	Significance
TET-1	WX♀(8) ×TK_{tet}♂(5)	8	781	701	0.905±0.025	TET-1:TET-2	*P*<0.0001(t=33.746 df=13)
TET-2	WX♀(8) ×TK♂(5)	7	688	0	0.000±0.000	TET-1:TET-3	NS(t=0.401 df=12 *P*=0.558)
TET-3	WX♀(8) ×WX♂(5)	6	589	508	0.874±0.051		
TET-4	TK♀(15) ×WX_{tet}♂(3)	7	811	675	0.834±0.044	TET-4:TET-5	*P*<0.0001(t=20.150 df=13)
TET-5	TK♀(15) ×WX♂(3)	8	828	0	0.000±0.000	TET-4:TET-6	NS(t=-0.736 df=13 *P*=0.758)
TET-6	TK♀(15) ×TK♂(3)	8	872	763	0.857±0.057		

* Numbers in parentheses refer to the numbers of mosquitoes used in the respective combinations.

For each cross, hatching rate value is expressed as mean± standard error. NS, nonsignificant *P*-value.
